# Time and Tissue Windows in Futile Reperfusion after Ischemic Stroke

**DOI:** 10.14336/AD.2024.1024

**Published:** 2025-10-21

**Authors:** Zhe Cheng, Hongrui Wang, Xiaokun Geng, Gary B. Rajah, Omar Elmadhoun, Guangge Peng, Yuchuan Ding

**Affiliations:** ^1^Department of Neurology and the Stroke Intervention & Translational Center (SITC), Beijing Luhe Hospital, Capital Medical University, China.; ^2^Luhe Institute of Neuroscience, Capital Medical University, Beijing, China.; ^3^Department of Neurosurgery, Wayne State University School of Medicine, Detroit, MI, USA.; ^4^Department of Neurosurgery, Munson Medical Center, Traverse City, MI, USA.; ^5^Division of Critical Care Medicine, Mayo Clinic, Rochester, MN, USA.

**Keywords:** vessel recanalization, non-rescue ischemic injury, reperfusion-induced injury, no-reflow phenomenon, functional prognosis

## Abstract

Reperfusion strategies such as vascular thrombolysis and thrombectomy are the first-line treatments recommended for acute ischemic stroke. However, only half of these patients achieve functional independence after endovascular reperfusion of large vessel occlusions. Timely restoration of blood flow is crucial, but not all reperfusion results in benefit, a phenomenon termed futile reperfusion. Futile reperfusion occurs when brain tissue has already suffered irreversible damage before reperfusion or when other factors undermine the benefits of restored blood flow. These factors include reperfusion-not rescued injury, reperfusion-induced injury, and the no-reflow phenomenon. The success of reperfusion therapies also hinges on timing and tissue condition after stroke. Defining these time and tissue windows more precisely could refine stroke interventions, potentially expanding effective reperfusion opportunities tailored to individual patients, thereby reducing the incidence of futile reperfusion. This perspective article delves into the complexities of futile reperfusion and the critical roles of time and tissue windows in determining stroke outcomes.

## Introduction

As a leading global cause of disability, ischemic stroke presents significant challenges to clinicians and researchers in optimizing therapeutic strategies. Reperfusion strategies such as thrombolysis and thrombectomy, which aim to restore blood flow, are key to improving outcomes. However, not all reperfusion efforts prove beneficial.

*Reperfusion, or recanalization,* refers to the reopening of an occluded blood vessel. Despite successfully opening the blocked vessel, adequate reperfusion is not always achieved, and the brain tissue may not receive sufficient blood flow resulting in limited neurological improvement. This can be attributed to futile reperfusion or the no-reflow phenomenon, caused by factors such as microvascular damage [1], inadequate downstream perfusion [2, 3], and complications like micro-emboli released during thrombectomy, or tissue edema [4].

In *Futile reperfusion,* blood flow is restored to the ischemic area of the brain but does not result in meaningful clinical improvement. This may occur if the brain tissue has already suffered irreversible damage before reperfusion is achieved or if other factors negate the potential benefits of restored blood flow [4, 5].

*The no-reflow phenomenon* adds complexity to the management of ischemic stroke. It occurs when sufficient blood flow is not restored to the microvasculature within the affected brain tissue, despite successfully reopening a blocked large vessel. Addressing the no-reflow phenomenon is essential to safeguarding the microvasculature and ensuring effective reperfusion at the cellular level. Understanding and mitigating the causes of futile reperfusion and the no-reflow phenomenon is vital for enhancing the efficacy of reperfusion therapies and improving clinical outcomes for stroke patients.

Importantly, Patient-Reported Outcomes (PROs) in Reperfusion Therapy is also the key to evaluate futile reperfusion [6]. While clinical measures such as recanalization rates, infarct size, and functional scales like the modified Rankin Scale (mRS) provide quantitative insights into the success of reperfusion therapies, they often fail to capture the subjective experience of patients. PROs add a qualitative dimension that enhances our understanding of how patients perceive their recovery, functionality, and quality of life post-reperfusion. Some patients report significant cognitive decline, persistent fatigue, or emotional distress, regarded as Symptom Burden, despite successful reperfusion. These outcomes are often underrepresented in clinical assessments, but can be considered part of futile reperfusion from the patient's perspective. PROs such as fatigue scales, cognitive function tests, and mental health assessments can reveal more subtle deficits that significantly affect a patient’s long-term recovery and well-being. Using tools like the Stroke-Specific Quality of Life (SS-QoL) scale can help capture a broader array of outcomes that matter to patients, including independence, mobility, and emotional stability [7]. Patients who achieve successful reperfusion but continue to struggle with daily activities may view their treatment as less effective, further expanding the definition of "futile" reperfusion. Finally, from a patient’s viewpoint, functional independence in daily living activities is often a key measure of recovery. Incorporating patient feedback through the Barthel Index or other functional independence measures may reveal that many patients do not return to their pre-stroke baseline in terms of independence, despite positive imaging or clinical scores, thus rendering the reperfusion less meaningful [8, 9]. Taken together, by integrating PROs, we can better understand and reduce the factors that contribute to futile reperfusion, thus making stroke interventions to improve both clinical outcomes and patient satisfaction.

**Figure 1.**
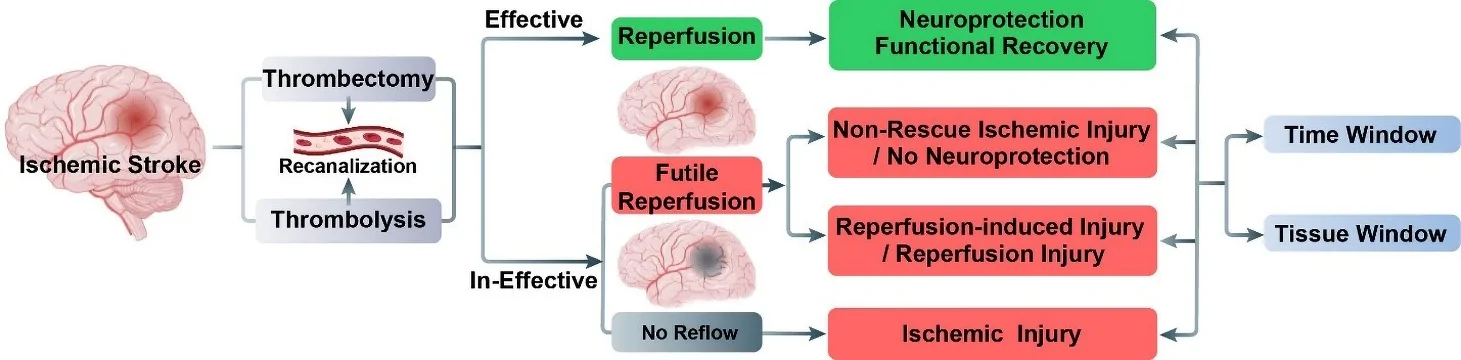
**Reperfusion After Ischemic Stroke**. This figure illustrates the fate of reperfusion strategy following ischemic stroke. Despite the successful reopening of an occluded vessel, the desired clinical outcomes may not be achieved due to inadequate reperfusion. This includes non-rescue ischemic injury, reperfusion-induced injury, and the non-reflow phenomenon, all of which can contribute to poor clinical outcomes. Futile reperfusion refers to scenarios where attempts to restore blood flow to salvage ischemic brain tissue fails or even exacerbates tissue damage. In the no-reflow phenomenon, even with a reperfusion strategy, adequate blood flow is not restored to brain tissue that was deprived of oxygen and nutrients during the ischemic event. Consequently, the tissue may not recover despite timely restored blood flow due to irreversible damage sustained during the ischemic period, emphasizing the critical role of appropriate intervention within the optimal "time window" and the variable "tissue window" of ischemic tolerance.

## Reperfusion: More than Just Blood Flow Restoration

Following successful reperfusion, there are two potential outcomes: effective and ineffective (Fig. 1). Effective reperfusion leads to neuroprotection and functional recovery, often achievable when therapeutic intervention occurs within the optimal *time window* after stroke onset. In contrast, ineffective reperfusion results in futile reperfusion characterized by reperfusion-not rescued injury and/or reperfusion-induced injury, as well as the no-reflow phenomenon.

## Reperfusion-Not Rescued Injury

Reperfusion-not rescued injury occurs where the restoration of blood fails to salvage ischemic tissue. If the tissue has already reached an irreversible point before reperfusion, restoring blood flow cannot revive necrotic cells or reverse extensive cellular damage. Therefore, the timing of reperfusion is crucial, as delayed reperfusion diminishes the potential for tissue salvage [10]. Beyond this “time window”, the likelihood of rescuing the tissue decreases significantly, resulting in permanent damage and deficits.

Individual tolerance to ischemic insults also plays a role [11]. Brain tissue in certain individuals may be inherently more vulnerable to ischemia, experiencing rapid and irreversible damage, while others may withstand longer periods of ischemia with less severe consequences due to variability in cellular, vascular capacity and collateral circulation [12]. This concept of a "tissue window" reflects the heterogeneity in ischemic resistance among different brain regions and patients. Magnetic Resonance Imaging (MRI) techniques, such as diffusion-weighted imaging (DWI) and perfusion-weighted imaging (PWI), play a role in identifying the ischemic penumbra, the area of brain tissue at risk but potentially salvageable with timely reperfusion therapy. While MRI mismatch provides valuable insights into brain tissue viability and guides therapeutic decisions, it does not definitely predict outcome brain injury following reperfusion therapy after ischemic stroke [13]. Not all patients with mismatch achieve favorable outcomes post-reperfusion [14], while there are also reports of DWI reversal after reperfusion [15]. A recent *JAMA* study suggests that thrombectomy may be beneficial for large core strokes, regardless of mismatch areas, indicating that mismatch does not solely determine outcomes after thrombectomy and reperfusion [13]. Additionally, a *Stroke* study further indicated that successful reperfusion after thrombectomy correlates with a good prognosis even in patients without mismatch, challenging the notion that unsalvageable tissue determined by computed tomography perfusion (CTP) accurately predicts good outcomes [14].

Taken together, both the "time window" and "tissue window" are crucial in understanding reperfusion-not rescued injury and may result from a complex interplay involving timing, individual patient characteristics, and additional pathophysiological processes. While reperfusion is critical, it does not guarantee complete resolution. This underscores the necessity for a comprehensive approach to ischemic stroke treatment, incorporating timely intervention and an understanding of individual tissue responses.

## Reperfusion-Induced Injury (Reperfusion Injury)

Reperfusion-induced injury, also commonly known as ischemia-reperfusion injury, occurs when the restoration of blood flow (reperfusion) to ischemic tissue. This restoration can lead to additional damage beyond that caused by the initial lack of oxygen and nutrients (ischemia). This paradoxical phenomenon arises because the sudden return of blood flow brings a surge of oxygen and blood cells resulting in several detrimental effects. First, the sudden influx of oxygen generates reactive oxygen species, which can damage cellular components such as DNA, proteins, and lipids [16]. Second, it triggers an inflammatory response, leading to further tissue damage and cellular death [17, 18]. Third, it disrupts cellular calcium homeostasis, causing further cellular injury or death [19]. Fourth, it breaks down the blood-brain barrier (BBB) [20-22]. And fifth, it damages the mitochondria resulting in reduced energy production and cell viability [23].

Reperfusion can activate various molecular pathways that promote apoptosis, necrosis, and other forms of cell death. Additionally, reperfusion strategies, especially if performed late, may cause brain edema and intracranial hemorrhage, leading to poor prognosis [24].

**Figure 2.**
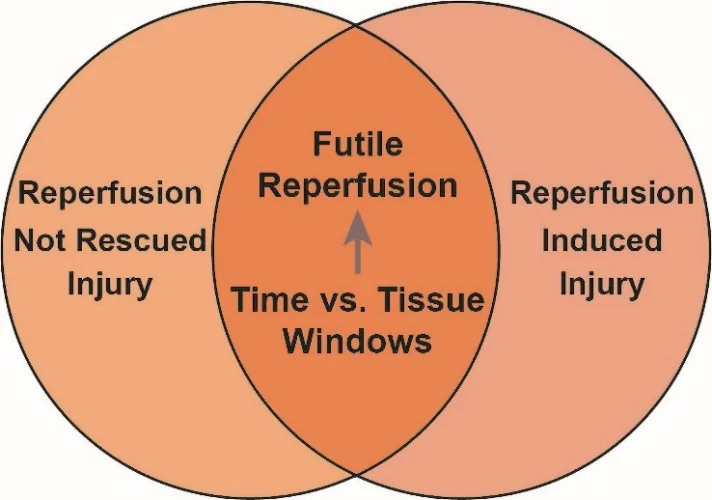
**Futile Reperfusion with Reperfusion-Not Rescued Injury and Reperfusion-Induced Injury**. This diagram visually represents the overlapping nature of two major types of injury following reperfusion, the Reperfusion-Not Rescued Injury and Reperfusion-Induced Injury. Both injuries occur after the restoration of blood flow to previously ischemic brain tissue. The interaction of the "Time Window" and "Tissue Window" is crucial in determining the extent of futile reperfusion, where reperfusion therapy fails to provide therapeutic benefit, leading to futile reperfusion with ongoing tissue damage despite successful restoration of blood flow.

## Relationship of Reperfusion-Not Rescued and Reperfusion-Induced Injuries

Reperfusion-not rescued injury and reperfusion-induced injury share some similarities and may interact (Fig. 2). Both occur after the restoration of blood flow (reperfusion) to previously ischemic brain tissue and represent challenges and complications in the treatment of ischemic stroke. They reveal overlapping features, such as the potential for continued cell death and neurological damage despite successful reperfusion. However, reperfusion-not rescued injury occurs when reperfusion fails to salvage the ischemic tissue, while reperfusion-induced injury (reperfusion injury) happens when the restoration of blood flow itself causes additional damage. Both types of injury contribute to futile reperfusion.

Reperfusion-not rescued and reperfusion-induced injuries can occur simultaneously to varying degrees in the same patient or some tissues, and the boundaries between these types of injuries can be indistinct. The critical "time window" and "tissue window" may play pivotal roles in determining the extent and nature of these injuries. Addressing the complex interplay between these injuries requires a comprehensive approach, including advanced imaging techniques and biomarkers to identify vulnerable tissues. It should also incorporate neuroprotective strategies to shield neurons from reperfusion-induced damage, and personalized treatment plans to optimize recovery based on individual patient profiles [25-27].

## No-Reflow Phenomenon

**Figure 3.**
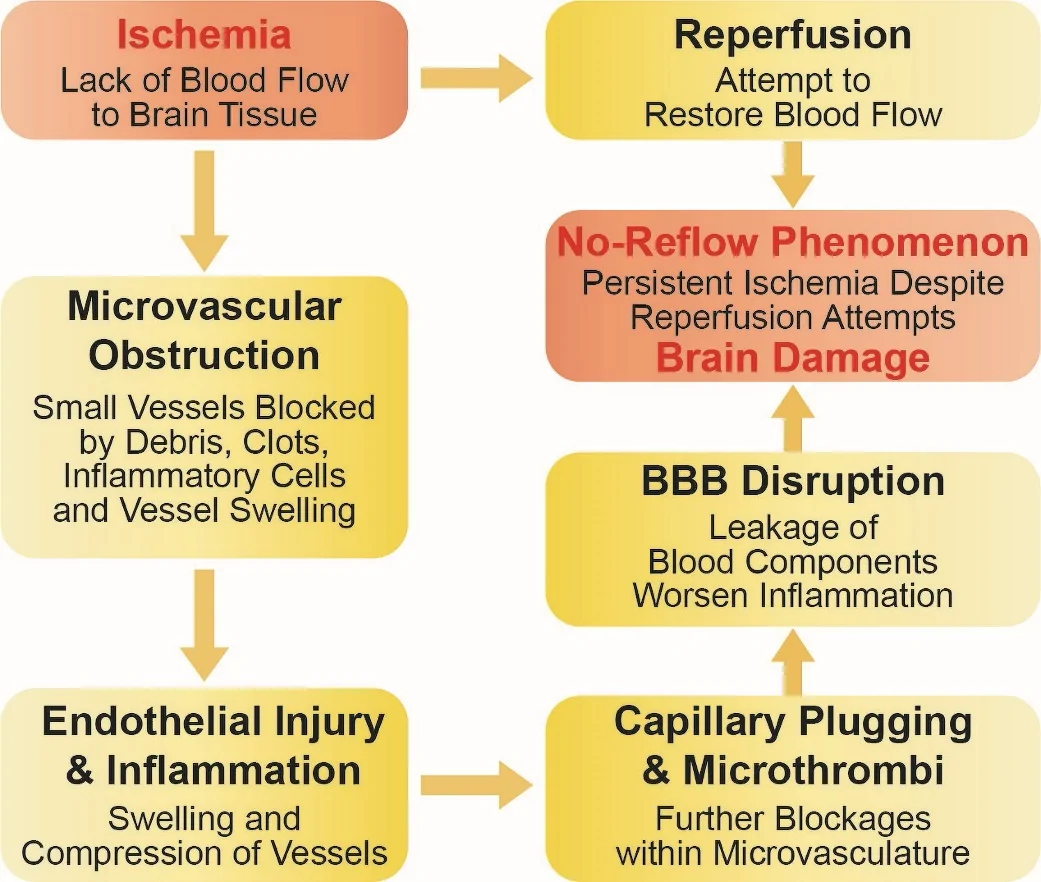
**No-Reflow Phenomenon**. The diagram illustrates the cascade of events leading to the no-reflow phenomenon following ischemia and attempted reperfusion. It highlights how microvascular obstruction, caused by debris, blood clots, inflammatory cells, and vessel swelling, leads to persistent ischemia. Endothelial injury and inflammation further exacerbate microvascular spasm and compression, while BBB (Blood-Brain Barrier) disruption worsens inflammation through blood component leakage. Additionally, capillary plugging and microthrombi formation contribute to impaired blood flow, even after the main vessel has been cleared, ultimately resulting in brain damage and suboptimal neurological recovery post-reperfusion therapy.

The no-reflow phenomenon is primarily caused by microvascular obstruction, where small blood vessels (capillaries/microcirculation) within the affected brain tissue remain blocked. This blockage occurs due to the accumulation of debris, blood clots, inflammatory cells or swelling of the vessel walls. This prevents adequate blood flow and oxygen delivery to all affected areas [28, 29] (Fig. 3).

Endothelial injury during the ischemic period can damage blood vessels, leading to dysfunction and inability to regulate blood flow properly [30, 31]. Ischemia and reperfusion can cause inflammation [32], resulting in swelling and compression of microvessels, further impeding blood flow [33]. Microvascular spasm [34] and endothelial swelling [35] can also contribute to the no-reflow phenomenon. Tissue swelling compresses blood vessels, reducing the effectiveness of reperfusion [33]. Other factors involved in the no-reflow phenomenon include capillary plugging by escaped microemboli and debris [36], or microthrombi formation where small blood clots form within the microvasculature, blocking blood flow even after the main vessel has been cleared [37]. In addition, BBB disruption causes leakage of blood components into the brain tissue, exacerbating swelling and inflammation [38-40]. Consequently, patients may not experience the expected neurological recovery after reperfusion therapy due to persistent ischemia in affected areas.

## Understanding the Time and Tissue Window

The "time window" refers to the critical period after a stroke during which therapeutic interventions must be initiated to achieve effective reperfusion. Traditionally, this window for intravenous thrombolysis is within 4.5 hours of stroke onset, based on the pharmacokinetics of thrombolytic agents like alteplase [41]. Recent research suggests that for some patients, this window might be extended up to 24 hours [42]. Similarly, for thrombectomy, this window can also be extended up to 24 hours [43]. However, this time window may vary significantly among individuals, influenced by the “tissue window”.

The "tissue window" reflects the cellular and vascular dynamics that determine the viability of the ischemic penumbra. This window describes the physiological and cellular state of the ischemic penumbra in its ability to recover once blood flow is restored. Factors affecting the tissue window include both cellular and vascular factors, such as the metabolic demand of the affected tissue [44], the degree of collateral blood flow during the ischemic event [45], and intrinsic cellular stress response [46]. A narrow tissue window indicates rapid progression toward irreversible damage, making even timely reperfusion ineffective if cellular recovery mechanisms are too compromised. Additionally, gene expression influences the tissue window by affecting the brain's response to ischemia and reperfusion, further determining recovery capacity [47, 48]. The relationship between the time and tissue windows is dynamic and crucial for successful reperfusion therapy (Figure 1). The "time window" provides a general guideline based on population-wide data, while the "tissue window" is individualized, reflecting each patient's unique response to ischemic injury. Variability in susceptibility to ischemia among patients and within different brain regions emphasizes the need for personalized treatment approaches.

Optimal treatment outcomes depend not only on recognizing the appropriate time window but also on understanding the state of the brain tissue within that timeframe. Effective reperfusion requires intervention within a period when the ischemic tissue still has the capacity to recover, aligning the time and tissue windows. Misalignment due to delayed intervention or rapid cellular damage progression can result in futile reperfusion and poor clinical outcomes.

Advancing our understanding of the mechanisms that influence these windows through enhanced imaging techniques, gene detection, and biomarkers could significantly improve the precision and effectiveness of stroke interventions. However, many centers, especially in less-resourced areas, lack access to advanced imaging technologies like MRI or perfusion CT and rely instead on basic techniques such as ASPECTS (Alberta Stroke Program Early CT Score) and CT scans. This reliance on generalized time windows can lead to suboptimal treatment decisions for patients whose tissue windows may require a different approach.

## Potential Strategies to Mitigate Futile Reperfusion

The phenomenon of futile reperfusion highlights the need for early intervention in ischemic stroke management. Rapid and effective reperfusion therapies are essential for maximizing the possibility of tissue recovery. Addressing both macrovascular and microvascular reperfusion is crucial; ensuring adequate perfusion at the microvascular level can help mitigate continued ischemia and cell death, even after large vessel reperfusion.

Understanding mechanisms, including gene expressions, which drive individual variability in tissue resistance and response to ischemia can lead to more personalized treatment approaches. Identifying patients at higher risk of reperfusion-not rescued injury can also help tailor interventions to improve outcomes. Recognizing and managing the no-reflow phenomenon through careful monitoring during and after reperfusion therapy is vital. Treatments aimed at protecting the microvasculature and ensuring adequate perfusion can help mitigate the effects of no-reflow. These therapies, when used alongside reperfusion strategies, can enhance tissue recovery and functional outcomes. Enhancing neuroprotection may involve extending the therapeutic window through neuroprotective agents that stabilize cellular metabolism and reduce ischemic injury [26, 49, 50]. Advanced imaging techniques and biomarkers could help tailor interventions to individual pathophysiological timelines, potentially extending the time window for patients. Additionally, identifying key genetic targets and developing therapies that modulate gene expression could optimize the “tissue window” for better recovery.

## Emerging Therapies and Technologies Affecting Effectiveness of Reperfusion

Recent advances in neuroprotection have focused on agents that can mitigate the damage caused by ischemia and reperfusion. These agents aim to protect neurons from oxidative stress, inflammation, and apoptosis, all of which are exacerbated during reperfusion. N-Acetylcysteine (NAC) is known for its antioxidant properties. NAC has shown potential in reducing oxidative damage during ischemic stroke and improving neuronal survival post-reperfusion [51]. Minocycline is an anti-inflammatory agent that has been demonstrated to reduce microglial activation and attenuate the inflammatory response during stroke. Studies suggest that minocycline may provide benefits when administered alongside reperfusion therapies [52]. Edaravone, a free radical scavenger approved in some countries for stroke treatment, works by reducing oxidative stress during reperfusion and improving outcomes in patients undergoing thrombolytic therapy [53].

Thrombolytic drugs for thrombolysis are a critical part of reperfusion therapy, and newer thrombolytic agents are emerging as alternatives to traditional drugs like alteplase (tPA). Tenecteplase (TNK-tPA) is a promising thrombolytic agent due to its superior fibrin specificity, longer half-life, and ease of administration compared to alteplase [41, 54, 55]. Clinical trials have shown that tenecteplase may be just as effective, if not more so, in achieving successful reperfusion while reducing the risk of hemorrhage. Desmoteplase, derived from vampire bat saliva, has been investigated for its ability to selectively target fibrin-rich clots, offering the potential for a safer thrombolytic profile, particularly in late-presenting stroke patients [56].

In the territory of mechanical thrombectomy, rapid advancements in Innovative Surgical Techniques have significantly improved reperfusion success rates. Stent Retrievers, which are the devices like the Solitaire and Trevoretrievers, have revolutionized mechanical thrombectomy by mechanically removing clots from large arteries [57, 58]. They have been shown to improve recanalization rates and patient outcomes, particularly when used in conjunction with thrombolysis. Aspiration thrombectomy has emerged as a complementary or alternative technique to stent retrievers [59, 60]. Devices such as the Penumbra system utilize suction to remove the clot and have shown high rates of complete revascularization [61]. In addition, the growth of techniques combining aspiration and stent retrieval (Solumbra technique) offers promising results in optimizing clot removal, reducing procedure times, and improving functional outcomes in patients [62].

Taken together, incorporating these innovations into stroke management could significantly enhance the efficacy of reperfusion therapies and reduce the incidence of futile reperfusion.

## Pharmacology, Biotechnology, and Healthcare Policy in Reperfusion Strategy

Recent advancements in pharmacology have introduced new avenues for improving reperfusion outcomes in ischemic stroke. For example, the development of next-generation thrombolytic drugs that aim to extend the time window for thrombolysis with better safety profiles is a significant pharmacological advancement. As mentioned above, the agents like tenecteplase are currently being investigated as alternatives to alteplase, offering longer half-lives and greater fibrin specificity, which may reduce the risk of hemorrhagic complications [54]. Beyond thrombolysis, the role of neuroprotective pharmacological agents is being explored to mitigate reperfusion-induced injury. Drugs targeting excitotoxicity, oxidative stress, and inflammatory pathways, such as NMDA receptor antagonists or free radical scavengers, are showing potential in preclinical studies [63]. However, translating these pharmacological advancements into clinical practice remains challenging due to the complexity of ischemia-reperfusion mechanisms.

Biotechnology is playing an increasingly important role in the treatment and management of ischemic stroke. Innovations such as advanced clot retrieval devices (e.g., stent retrievers and aspiration catheters) are improving mechanical thrombectomy outcomes, especially in patients with large vessel occlusions [64]. Additionally, advances in imaging technologies such as perfusion MRI and CT, combined with artificial intelligence (AI)-based analysis, allow for better identification of the "tissue window" [65]. This enables more personalized treatment strategies, ensuring that patients receive appropriate interventions based on the real-time status of ischemic brain tissue. Moreover, biotechnology companies are working on developing biomaterials and tissue-engineered scaffolds that can be used to promote neural regeneration after stroke [66], offering hope for enhanced recovery even in patients who have experienced significant tissue damage.

Healthcare policy plays a critical role in ensuring equitable access to stroke treatment. One challenge that remains is the inconsistent availability of advanced stroke care, including endovascular thrombectomy, across healthcare systems. Policies that support the development of comprehensive stroke centers and improve inter-hospital transfer protocols can ensure timely access to reperfusion therapies, ultimately reducing the burden of futile reperfusion.

In addition, policies aimed at increasing funding for stroke research and facilitating clinical trials for emerging treatments are essential for translating scientific discoveries into clinical practice. Moreover, implementing policies that promote the use of telemedicine in acute stroke care can help to overcome geographic barriers and bring specialized care to underserved areas, reducing the time to treatment and improving patient outcomes.

Taken together, by integrating insights from pharmacology, biotechnology, and healthcare policy, we are better able to provide a more comprehensive understanding of the multifaceted challenges associated with ischemic stroke treatment. The interplay of these disciplines highlights how advancements in each area contribute to overcoming the limitations of current reperfusion strategies and ultimately improving patient outcomes.

## Conclusion and Future Directions

Futile reperfusion in ischemic stroke underscores the importance of understanding both the “time window” and “tissue window” for effective therapy. Therefore, the success of reperfusion treatments hinges on timing and tissue viability, including neuronal sensitivity and collateral circulation. Addressing the challenges associated with futile reperfusion, no-reflow phenomenon and individual patient factors is essential for improving clinical outcomes and enhancing therapeutic efficacy.

Future research must focus on personalizing stroke treatment by leveraging advanced imaging, biomarkers and gene detection to more accurately define the optimal time and tissue windows for each patient. Innovations in neuroprotective agents, along with a deeper understanding of tissue sensitivity and variability, have the potential to find more effective and targeted therapies. In addition, recognizing the limitations of reperfusion and understanding the interplay of timing and tissue health are imperative. Advancing our knowledge will pave the way for more personalized interventions, ultimately improving recovery and quality of life for stroke patients.
